# Editorial: The association between avian physiology and meat quality

**DOI:** 10.3389/fphys.2024.1368680

**Published:** 2024-01-31

**Authors:** Yuwares Malila, Marco Zampiga, Francesca Soglia, Casey M. Owens, Sandra G. Velleman

**Affiliations:** ^1^ National Center for Genetic Engineering and Biotechnology (BIOTEC), Pathum Thani, Thailand; ^2^ Department of Agricultural and Food Sciences, Alma Mater Studiorum, University of Bologna, Cesena, Italy; ^3^ Department of Poultry Science, University of Arkansas, Fayetteville, AR, United States; ^4^ Department of Animal Sciences, The Ohio State University, Wooster, OH, United States

**Keywords:** nutrition, meat, chicken, turkeys, ducks, muscle

## Introduction

World population is projected to reach 9 billion by the year 2050. With restricted availability of natural resources, food shortage, particularly of protein, has become a global concern (FAO, 2009). A variety of food protein alternatives have been recently developed and launched to the market. However, during the next decades, a rise in poultry meat demand has been projected worldwide (USDA, 2023). This is due to its excellent nutritional value, high production efficiency, affordable price for low-income families and lesser greenhouse gas emissions than other livestocks. The poultry meat produced must be of high quality to ensure food security and minimize food waste.

It is widely accepted that meat quality is tightly linked with animal physiology (Joo et al., 2013; Terlouw et al., 2021). Although several studies have previously documented such a fundamental link, several factors such as the dramatic change in climate conditions due to global warming and the occurrence of muscle abnormalities may exert stress to avian species in different ways than previously experienced. On the other hand, the increasing availability of newly-developed farm management tools and feed additives can help to maintain optimal physiological state and meat quality even in such challenging conditions. The ultimate goal of this Research Topic: *The Association between Avian Physiology and Meat Quality* was to provide comprehensive updates on such aspects and extend our understading to aid in assuring a sustainable production of high-quality poultry meat.

As a consequence of selection for productive traits, commercial meat-type chickens and turkeys exhibit rapid growth and relevant accretion of muscle mass, particularly of the breast muscle. In this Research Topic, Lee et al. compared the histological characteristics of myofibers and muscle bundles in *pectoralis major* and *gastrocnemius* muscles of commercial broilers and layers. The clear histological differences between the two breeds suggested that the greater muscle mass of fast-growing meat-type chickens could be a result of both myofiber hyperplasia and hypertrophy. Using male Japanese quails as a model, Kim et al. demonstrated the role of myostatin (*MSTN*) gene on histological characteristics and composition of breast muscle. A significant increase in body weight and muscle mass was observed in the *MSTN* knock-out quails with no differences in meat quality indices compared to the wild-type. A slight but significant increased proportion of glycolytic fast-twich (type IIB) fibers was observed in the deep region of the breast collected from the knock-out birds without any impacts on *postmortem* pH of the breast meat. This research report suggested the potential application of *MSTN* mutation for enhancing muscle mass of the birds while determining no significant effects on meat quality.

On the other hand, breeding selection focusing mainly on production performance appeared to exert a negative impact on poultry meat quality. The massive muscling, focusing on breast muscles, of fast-growing birds appeared to outgrow their life support systems, particularly vascularization, leading to development of growth-related myopathies. As reported in an opinion article of Malila, growing evidence has indicated an association between growth-related myopathies and *in-vivo* oxidative stress, which in its turn can impair meat quality because of lipids and proteins oxidation. Several feed additives having antioxidant activities have been examined for improving meat quality. Herein, the effects of tannic acid were addressed by Choi et al.. Results showed that the dietary supplementation of tannic acid up to 2 g/kg appeared to negatively affect overall growth performance, feed efficiency, bone health and fat accumulation. However, tannic acid supplementation in starter/grower phases enhanced gut health and nutrient transportation, and increased nutrient digestibility in the finisher phase. These findings not only supported the benefits of tannic acid on gut health but also suggested the dosage and the optimal duration of the supplementation.

In terms of flavor, the meat belonging to fast-growing birds appeared to contain a lower amount of inosine monophosphate (IMP), the most important umami compound in the meat. To define biological pathways associated with IMP deposition in chicken meat, Yu et al. compared the transcriptome profiles of chicken muscles showing relatively high or low IMP content. The authors investigated the effects of muscle tissue (breast vs leg), gender (hen vs rooster), production management (cage vs free-range) and growth rates (fast vs slow). Potential candidate genes regulating IMP muscle deposition were identified for further breeding program.

Interestingly, Zhang et al. reported a connection between knob size, one of the important consumer purchasing criteria in China, and bone protrusion size in Yangzhou geese. Despite no differences in production performance, leg muscle of geese with large knob exhibited a greater insoluble collagen and expressible water content along with a higher growth hormone levels than those of small-knob geese.

A large proportion of the articles in this Research Topic focused on the impact of thermal stress as climate change is one of the most urgent challenges affecting all living organisms. Cartoni Mancinelli et al. investigated the relationships among behavior, physiological conditions, and meat quality of commercial broilers exposed to chronic heat stress. They observed a two-stage behavioral response when the environmental temperature reached 25°C and over 27°C. The modified behaviors were associated with altered blood parameters reflecting an oxidative and inflammatory state that affected breast meat quality. Such findings offered crucial insights for identifing thermal discomfort among broilers as well as to better understand the impact of heat stress on meat quality. Reed et al. examined differential expression patterns of non-coding microRNAs (miRNAs) in turkey muscle stem cells (SCs) to define an in-depth biological response to thermal challenges. Potential target genes of differentially expressed miRNAs were also predicted to underline the potential consequences of the miRNA differential expression. Overall, their findings suggested a significant impact of thermal challenges on SCs proliferation and differentiation among the fast-growing birds. The crucial roles of SCs in muscle growth, development, repair and subsequent meat quality of poultry are further emphasized in an opinion article by Velleman. It was suggested that the assement of SCs biological activity upon thermal challenges should be included in the poultry selection strategies.

Nutritional strategies for heat stress alleviation were also extensively investigated. Herein, Brugaletta et al. studied the response of commercial broilers to arginine supplementation upon an exposure to cyclic thermal stress. Although arginine supplementation at the tested dosage did not significantly enhanced the productive performance of heat-stressed broilers, the metabolomic analysis unveiled the potential role of arginine in counterbalancing the adverse effects of such stressor on energy homeostasis mechanisms through increasing creatine levels and regulating AMP levels. An increase in digestion and absorption of dietary amino acids was also hypothesized, suggesting the additional benefits of arginine supplementation on improving intestinal health and function under heat stress. Moreover, Señas-Cuesta et al. addressed the effects of providing *Lippia origanoides* essential oils containing herbal betaine to commercial broilers subjected to cyclic thermal stress. The dietary inclusion of such oils had some beneficial effects on body weight gain, intestinal conditions and bone quality compared to heat-stressed chickens.

Overall, the articles published in this Research Topic provide insightful updates and extend our comprehension regarding the link between avian physiology and meat quality to ensure the future production of high-quality poultry meat.

## References

[B1] FAO (2009). “Global agriculture towards 2050,” in High level expert forum—how to feed the world in 2050 (Rome, Italy: FAO).

[B2] Joo S. T. Kim G. D. Hwang Y. H. Ryu Y. C. (2013). Control of fresh meat quality through manipulation of muscle fiber characteristics. Meat Sci. 95 (4), 828–836. 10.1016/j.meatsci.2013.04.044 23702339

[B3] Terlouw E. M. C. Picard B. Deiss V. Berri C. Hocquette J. F. Lebret B. (2021). Understanding the determination of meat quality using biochemical characteristics of the muscle: stress at slaughter and other missing keys. Foods 10 (1), 84. 10.3390/foods10010084 33406632 PMC7823487

[B4] USDA (2023). Livestock and poultry: world markets and trade. Glob. Mark. Anal. Available online: https://apps.fas.usda.gov/psdonline/circulars/livestock_poultry.pdf (accessed on November 30, 2023).

